# Early cost-effectiveness analysis of the U-Care Renal Platform

**DOI:** 10.1186/s12882-026-05289-y

**Published:** 2026-08-14

**Authors:** Attila Imre, György Tímár, Andrea Ancona, Francesca Alfieri, Enrico Perpignano, Bertalan Jászkuti, Balázs Nagy, Bertalan Németh, Zoltán Kaló

**Affiliations:** 1 Center for Health Technology Assessment, Semmelweis University, Budapest, Hungary; 2 Center for Pharmacology and Drug Research & Development, Semmelweis University, Budapest, Hungary; 3 Syreon Research Institute, Budapest, Hungary; 4 U-Care Medical S.r.l, Turin, Italy

**Keywords:** Economic evaluation, Headroom analysis, U-Care Renal Platform, Acute kidney injury, Artificial intelligence

## Abstract

**Background:**

Acute kidney injury (AKI) is a common and serious complication in intensive care unit (ICU) patients, with reported incidence ranging from 16% in general hospitalized cohorts to over 20% in some ICU populations, and it increases mortality, prolongs hospitalization, and worsens long-term renal outcomes. The U-Care Renal Platform (UCRP) is a CE-marked AI-powered software as a medical device that, through its Prevention Module via the AKIRA predictive model, predicts moderate-to-severe AKI 24 hours before onset supporting the early application of KDIGO-guided preventive care bundles. We conducted an early cost-effectiveness analysis of UCRP versus standard of care (SoC) and estimated its economic headroom in Italian intensive care.

**Methods:**

A decision tree (acute phase, 0-90 days) combined with a Markov cohort model (chronic phase, years 1-20) was built from the Italian healthcare payer perspective. The modelled cohort included 2,000 annual ICU admissions with a 40% AKI incidence. UCRP diagnostic accuracy was set at 90% sensitivity and 70% specificity based on internal manufacturer data, with the published Italian external validation (82.5%/83.2%) examined as a scenario. In true-positive patients, early KDIGO-guided treatment was modelled as an approximately 22% relative shift from higher to lower AKI severity stages. Costs were Italian-specific; long-term transition probabilities came from UK NICE HTA data. Costs and QALYs were discounted at 3% annually over a 20-year horizon. Deterministic (±20%), scenario, threshold, and probabilistic (10,000-iteration Monte Carlo) sensitivity analyses were performed.

**Results:**

In the primary analysis, UCRP was projected to be dominant: an incremental cost saving of €363.42 per patient (total discounted cost €38,059.95 with SoC versus €37,696.53 with UCRP) and an incremental gain of 0.049708 discounted QALYs (9.890 versus 9.940), giving a net monetary benefit (NMB) of €1,854.66 per patient at €30,000/QALY. Ninety-day mortality was projected at 13.72% (SoC) versus 13.29% (UCRP), corresponding to 8.6 additional survivors per 2,000 patients annually. In the probabilistic analysis, mean saving was €175.47 per patient (95% simulation interval -€1,500.72 to €2,016.66) and mean QALY gain was 0.048830 (95% simulation interval: -0.138081 to 0.172619); UCRP was cost-effective at €30,000/QALY in 78.3% of simulations. UCRP remained cost-saving and QALY-gaining across incidence, effect-size, and diagnostic-accuracy scenarios, with savings ranging from €135.93 to €601.21 per patient; the model projected cost saving above an AKI incidence of 4.84% and positive NMB above 1.05%. Results were most sensitive to ICU daily cost, AKI incidence, the 22% severity-shift assumption, and UCRP sensitivity.

**Conclusions:**

In the base case, the model projected UCRP to be dominant relative to SoC in Italian ICUs, with lower costs and higher QALYs driven by AI-based early AKI detection and guideline-directed treatment. These are conditional model estimates, not observed effects.

**Supplementary Information:**

The online version contains supplementary material available at https://doi.org/10.1186/s12882-026-05289-y.

## Introduction

Hospitalized patients in critical care units are exposed to several complications. Acute kidney injury (AKI) is a common and serious complication in critically ill patients. Reported incidence varies with population, definition, and ascertainment method: Havaldar et al. (2024) reported hospital-acquired AKI in 16.11% of a large critically ill cohort [1], while other ICU populations, particularly postsurgical, report incidences above 20% [2, 3]. AKI is associated with severe clinical consequences, including high mortality rates [1], and high economic impact [4] in the short-run due to prolonged ICU and hospital stay and in the long-run due to increased morbidity rates [5]. Earlier detection of AKI may facilitate the adoption of strategies to preserve renal function and prevent further progression of kidney disease. Currently, AKI diagnosis relies on a rise in serum creatinine levels and/or fall in urine output [6].

With the availability and connectivity of electronic health records (EHRs) and application of artificial intelligence tools in data processing, there is tremendous opportunity to develop real-time patient care data driven models to support clinical decision making. U-Care Renal Platform (UCRP) is an innovative CE-marked class IIb software as a medical device (MDR 2017/745) that, through its Prevention Module via the AKIRA predictive model, predicts moderate and severe AKI within ICUs and provide actionable decision support to improve adherence to clinical KDIGO guidelines for AKI prevention [7]. The Prevention Module of the Platform leverages AI-powered predictions to identify patients at risk of AKI stage 2 and 3 in the following 24 h, potentially improving ICU outcomes through early intervention. UCRP automatically, continuously and in real time analyzes patient level clinical data extracted from medical diagnostic and monitoring devices of ICU patients to predict the risk of a future episode of moderate or severe AKI, which allows health care professionals to intervene in a personalized manner based on the decision support provided by UCRP and based on international guidelines [8].

The predictive model used is capable of recognizing AKI earlier (24–72 h) from previously measured data, providing an opportunity (and time) for intervention, as validated in retrospective clinical studies [8]. By enabling the early and personalized implementation of clinical interventions according to KDIGO 2012 (Kidney Disease: Improving Global Outcomes) guidelines [9], UCRP has the potential to reduce the number of AKI patients and shift them to a better AKI stage, which may eventually reduce mortality, costs at the hospital and healthcare system levels.

A large systematic review and meta-analysis including over 16,000 patients demonstrated that structured implementation of KDIGO bundle interventions, comprising optimization of hemodynamics, avoidance of nephrotoxic drugs, tight monitoring of renal function, and GFR-based medication dose adjustment reduced the composite risk of major adverse kidney events (12.6% vs. 17.6%; OR 0.73), the incidence of moderate-to-severe AKI (OR 0.65), and the need for renal replacement therapy (OR 0.63) compared with usual care [10].

Early-phase health technology assessment (eHTA) provides a framework for evaluating the value of innovative technologies at a stage when full clinical evidence may not yet be available, using modelling approaches to project long-term clinical and economic consequences and inform both developers and payers [11–14]. Given the novelty of AI-based AKI prediction platforms and the absence of head-to-head randomized evidence for UCRP versus standard of care (SoC) in Italian ICUs, an early cost-effectiveness analysis is appropriate for this evaluation. This analysis addresses only the narrow economic dimension of value and does not constitute a full eHTA assessment.

The objective of this study is to explore the economic value of UCRP in Italian ICUs from the perspective of the Italian healthcare payer.

## Methods

### Patient population

The modelled population comprised adult patients admitted to Italian general ICUs (not restricted to a specific clinical indication), reflecting the real-world setting in which UCRP is deployed. The cohort starting age was 50 years. The cohort size was set at 2,000 annual ICU admissions. AKI incidence was assumed at 40% of all ICU admissions, consistent with data provided by U-Care Medical based on real-world experience in general Italian ICUs. This is a manufacturer-informed assumption for the intended general-ICU deployment setting, therefore its impact is examined in a dedicated incidence-scenario and threshold analysis.

Background prevalence of CKD at model entry, independent of AKI-related CKD, was set at 11.05%. AKI severity distribution among affected patients under SoC was: Stage 1, 68.7%; Stage 2, 19.4%; Stage 3, 5.95%; Stage 3 with renal replacement therapy (RRT), 5.95% [15]. All model parameters are reported in Supplementary Table S1 in the Appendix.

### Model structure

**Fig. 1:**
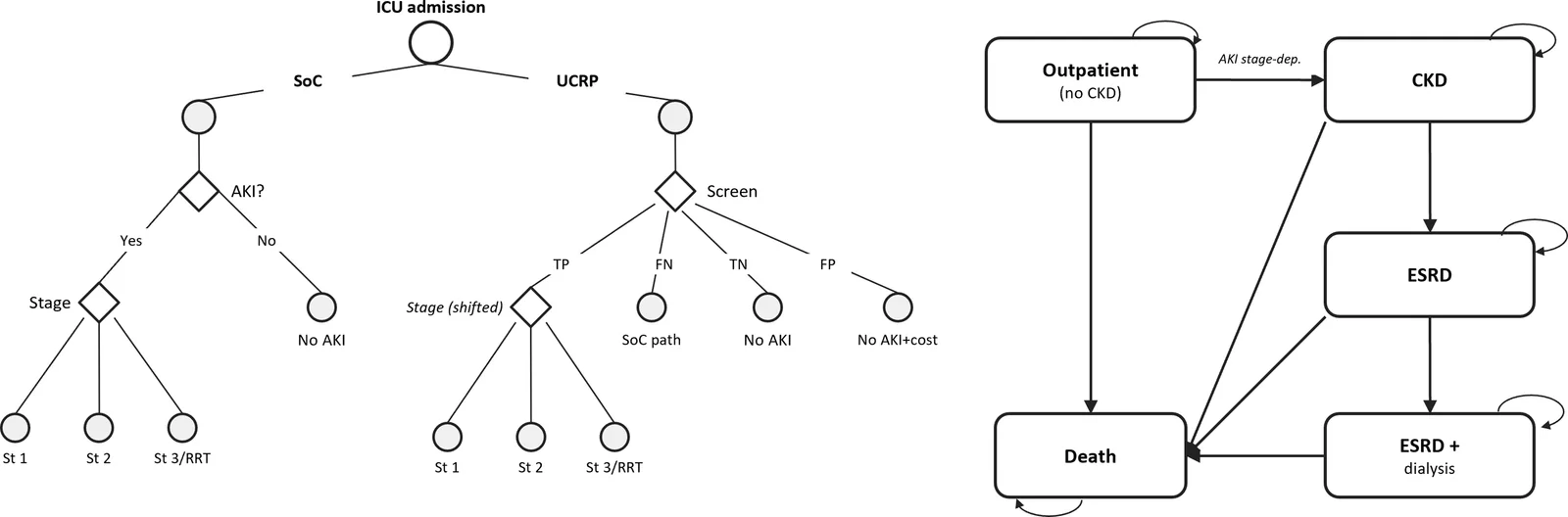
Model structure. Abbreviations: AKI, acute kidney injury; CKD, chronic kidney disease; ESRD, end-stage renal disease; FN, false negative; FP, false positive; ICU, intensive care unit; SoC, standard of care; TN, true negative; TP, true positive; UCRP, U-Care Renal Platform

Figure 1 depicts the model structure. A decision tree (acute phase, 0-90 days) combined with a Markov cohort model (chronic phase, years 1-20 with annual cycles) was developed in Microsoft Excel. The left side of Fig. 1 depicts the acute decision-tree (0-90 days). At ICU admission, patients are allocated to SoC or UCRP. Under UCRP, screening classifies patients into true positives (TP), false negatives (FN), true negatives (TN), and false positives (FP). TP patients receive early KDIGO-guided treatment and the modelled approximately 22% relative downstaging of AKI severity ("Stage (shifted)"). FN patients follow the SoC AKI pathway without early benefit. TN patients follow the no-AKI pathway. FP patients also follow the no-AKI clinical pathway but incur the modelled KDIGO-bundle cost. Each terminal node proceeds through the acute pathway (ICU → hospital ward → home) with stage-specific mortality. The right side of Fig. 1 depicts the long-term Markov phase (years 1-20, annual cycles). The surviving cohort (starting age 50 years) enters the Markov model in either the “CKD” or “No CKD” state. Then the cohort transitions between Outpatient (no CKD), CKD, ESRD, ESRD + dialysis, and Death (absorbing) states in annual model cycles, according to a set of transition probabilities [15]. Transition from Outpatient to CKD is AKI stage dependent.

The model was adapted from Brazzelli et al. (2022), with one modification which was the exclusion of renal transplantation [15]. Given the shortage of deceased donor kidneys in Italy [16], transplanting one patient necessarily extends the waiting and dialysis period for others; net system-level benefit from transplantation was therefore considered neutral and excluded from the analysis.

The model perspective is that of the Italian healthcare payer. The currency is EUR. The time horizon is 20 years. The willingness-to-pay (WTP) threshold was set at €30,000 per QALY, consistent with Italian HTA practice [17]. A one-way deterministic sensitivity analysis (DSA) was conducted by independently varying each model parameter by ± 20% from its base-case value. Scenario analyses crossed the AKI severity-shift effect (10%/22%/35%) with two diagnostic-accuracy cases (base-case; published Italian validation), and a threshold analysis identified the AKI incidence below which UCRP ceases to be cost-saving. A probabilistic sensitivity analysis (PSA, 10,000 Monte Carlo iterations) jointly simulated parameter uncertainty. Although the cost of UCRP was included in the base-case analysis, it is not disclosed for confidentiality reasons; therefore, a headroom analysis was used to illustrate the maximum price at which UCRP would remain cost-effective at the €30,000/QALY WTP threshold.

For presentation, per-bed results use a linear cost-allocation convention of 133.33 modelled patients per bed/year (2,000 admissions ÷ 15 beds).

### Diagnostic accuracy and clinical benefit

Diagnostic accuracy of UCRP was assumed to have 90% sensitivity and 70% specificity. These are internal manufacturer data. We include the published Italian external-validation accuracy (82.5% sensitivity, 83.2% specificity) as a scenario analysis [8]. Applied to the 2,000-patient cohort (800 AKI patients; 1,200 no AKI), this yields four screening sub-populations.

The clinical effect of UCRP in the TP arm was modelled as an approximately 22% relative shift from higher to lower AKI severity stages (from Stages 2 and 3 to stage 0 and 1), based on a single-centre before-and-after implementation study in which moderate/severe AKI occurred in 12/103 patients before and 9/99 after UCRP implementation [18]. As this study is small, postsurgical, and uncontrolled, it does not establish a causal effect; 10% and 35% scenarios are therefore also reported. FN patients follow SoC disease pathways. FP patients incur incremental KDIGO treatment costs.

### Cost and utility data

One day cost of ICU stay was €1,660, one day cost at a non-ICU hospital department was €475.86, the cost of single renal replacement therapy (acute dialysis) at an ICU was €128.95. Regarding long-term costs, annual cost of patients with chronic kidney disease (CKD) was €2,474, while annual cost of end-stage renal disease (ESRD) patients (if dialysis is not needed) was assumed to be €5,229.80 and annual cost of ESRD patients with dialysis was assumed to be €44,322.

Health-state utility values were sourced from the published literature. ICU utility was 0.4052; hospital ward utility 0.44; home utility 0.62 (applied uniformly across AKI stages in the acute phase, consistent with the source model). Long-term utility for the Outpatient state was 0.666, with disutility adjustments of -0.11 for CKD, -0.32 for ESRD, and − 0.41 for ESRD + dialysis. Background age-adjusted utility was applied throughout the Markov phase, declining from 0.938 (ages 0–29) to 0.650 (ages 85+).

### Transition probabilities

AKI-to-CKD annual conversion rates were stage-specific: 0.44% for No AKI, 0.88% for Stage 1, 1.32% for Stage 2, and 1.76% for Stage 3, an explicit 1×/2×/3×/4× structure anchored to the published 0.44% no-AKI baseline [15]. Long-term transition probabilities between Markov health states were sourced from the UK NICE HTA [15]; Italian-specific data of sufficient quality were not available to replace these inputs. Background mortality was age-adjusted throughout the model.

## Results

**Fig. 2:**
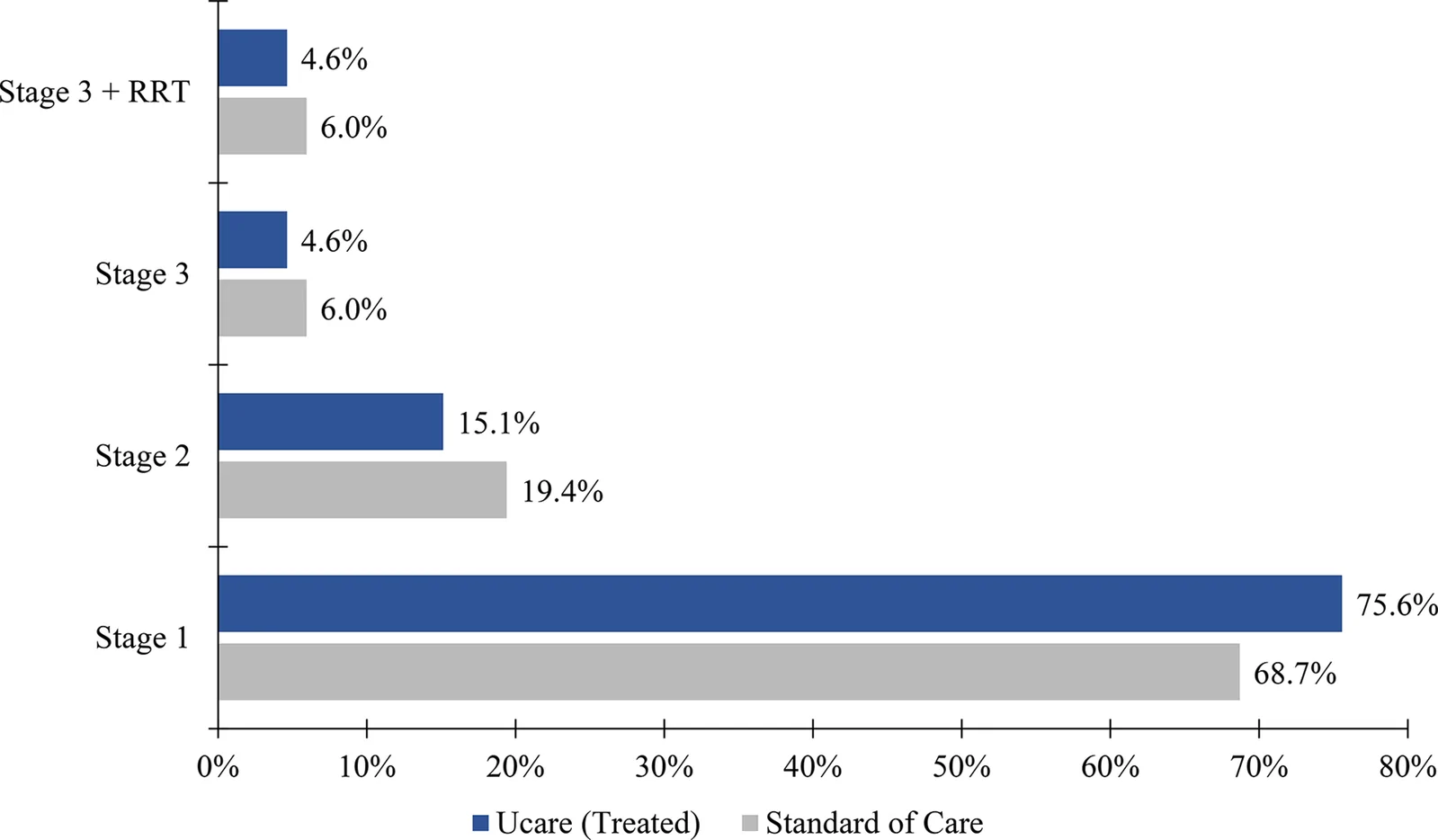
AKI severity distribution under SoC versus UCRP. Abbreviations: AKI, acute kidney injury; RRT, renal replacement therapy; UCRP, U-Care Renal Platform

In the modelled cohort of 2,000 patients (800 with AKI, 1,200 without, at the 40% incidence assumption), UCRP’s 90% sensitivity and 70% specificity classified 720 patients as TP, 80 as FN, 840 as TN, and 360 as FP. UCRP achieves its clinical benefit by shifting the AKI severity distribution in the true-positive arm toward less severe disease (Fig. 2). Under SoC, among patients with AKI, Stage 1 accounts for 68.7%, Stage 2 for 19.4%, Stage 3 for 5.95%, and Stage 3 + RRT for 5.95%. With the modelled UCRP effect, the distribution shifts to: Stage 1, 75.59% (+ 6.89% points); Stage 2, 15.13% (-22% relative); Stage 3, 4.64% (-22% relative); Stage 3 + RRT, 4.64% (-22% relative). This severity downstaging is the primary driver of the modelled cost and QALY differences.

The model projected a 90-day mortality rate of 13.72% under SoC and 13.29% under UCRP, an absolute difference of 0.43% points, corresponding to 8.6 additional survivors per 2,000-patient cohort per year. Total ICU days were projected at 8,435.2 (SoC) versus 7,897.6 (UCRP), a difference of 537.6 days (4.22 vs. 3.95 days per patient); ward days were projected at 15.89 (SoC) versus 15.76 (UCRP) per patient.

The model projected total discounted costs of €38,059.95 per patient with SoC and €37,696.53 with UCRP an incremental saving of €363.42. Discounted QALYs were 9.890 (SoC) and 9.940 (UCRP), an incremental gain of 0.049708. Incremental NMB was €1,854.66 at €30,000/QALY. UCRP was dominant (cost-saving and QALY-gaining) in the deterministic base case.

The incremental cost comprised €437.08 favoring UCRP for ICU care, €58.97 for hospital ward care, -€7.40 (favoring SoC) for home care, -€102.73 (favoring SoC) for long-term Markov-phase care (Table 1).

Dividing the 2,000-patient annual cohort by the illustrative 15-bed unit gives 133.33 modelled patients per bed/year. Under this convention, the model projects, per bed/year: 35.84 ICU days and 16.48 ward days saved (52.32 inpatient days in total); a cost saving of €48,455.47; a QALY gain of 6.627758; and an NMB of €247,288.20 at €30,000/QALY.

In addition to the base-case analysis above, we report the maximum justifiable price for UCRP per patient, which at a €30,000/QALY was €1,877.16 per patient.

Because the 40% AKI-incidence assumption is a manufacturer-informed input rather than published Italian epidemiology, its effect was examined directly. At incidences of 16.11%, 20%, 30%, and 40%, the model projects savings of €116.50, €156.70, €260.06, and €363.42 per patient, and NMB of €717.10, €902.33, €1,378.49, and €1,854.66. UCRP remains cost-saving above an incidence of 4.84% and NMB-positive above 1.05%.

Analyzing the severity-shift effect (10%/22%/35%) with two diagnostic-accuracy cases (internal base case 90%/70%; published Italian validation 82.5%/83.2%) produced combinations in which UCRP remained cost-saving and QALY-gaining throughout. Savings ranged from €135.93 (10% effect, published Italian accuracy) to €601.21 (35% effect, base-case accuracy) per patient; NMB ranged from €757.29 to €2,973.65. At the 22% effect with published Italian diagnostic accuracy, the model projected a saving of €337.14 and an NMB of €1,704.12 per patient.

**Fig. 3:**
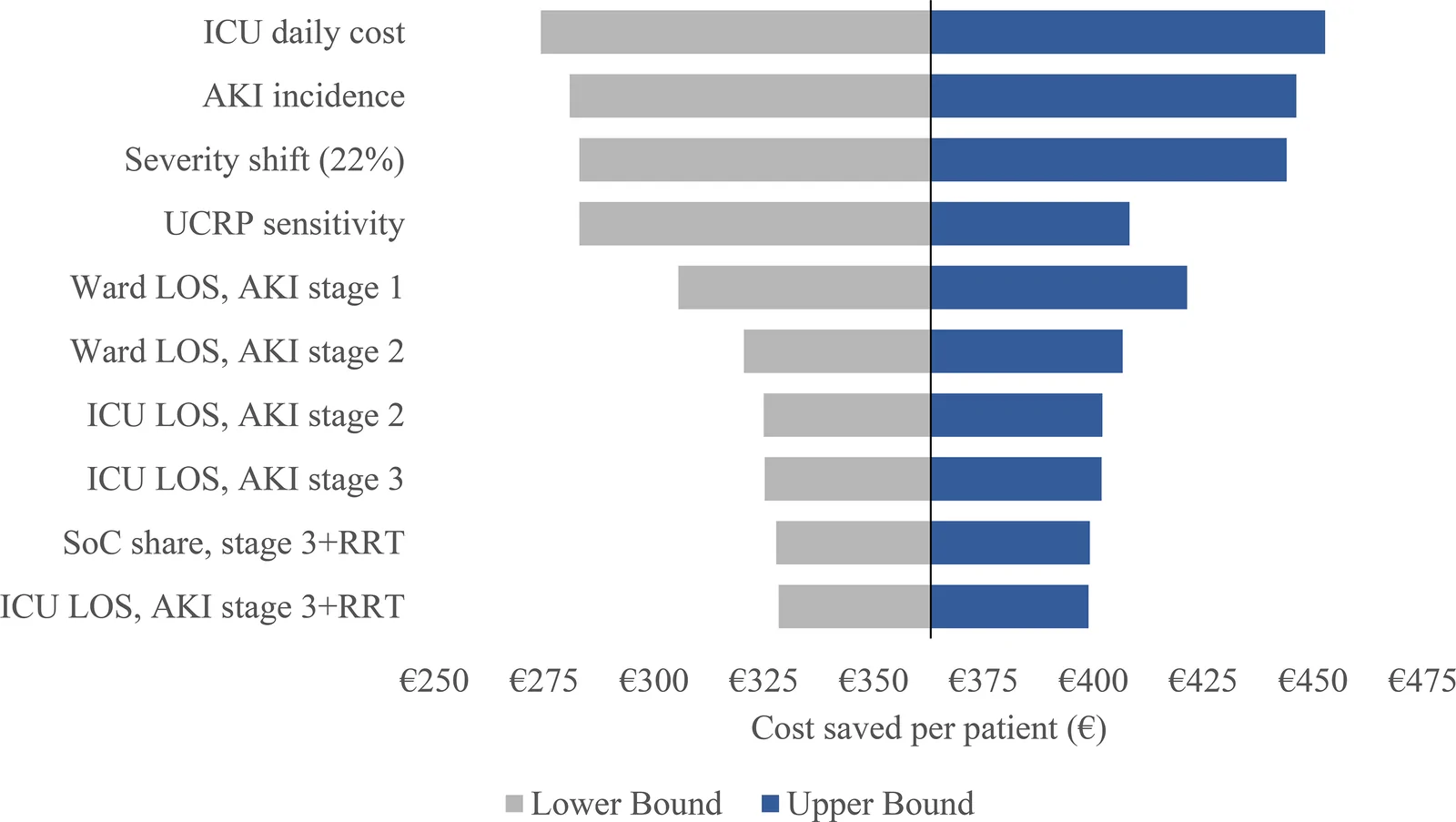
Tornado diagram. Abbreviations: AKI, acute kidney injury; ICU, intensive care unit; LOS, length of stay; SoC, standard of care; UCRP, U-Care Renal Platform

The one-way DSA (Fig. 3) varied each parameter by ± 20%, subject to natural bounds. The most influential parameters, ranked by their range of impact on cost saving per patient, were ICU daily cost, AKI incidence, the severity-shift effect, UCRP sensitivity and ward length of stay for AKI stage 1. No parameter, at either its upper or lower bound, reversed the finding that UCRP is cost-saving.

Across 10,000 Monte Carlo simulations (Supplementary Figure S1 and Supplementary Figure S2) mean incremental cost saving was €175.47 per patient (95% simulation interval -€1,500.72 to €2,016.66) and mean incremental QALY gain was 0.048830 (95% simulation interval − 0.138081 to 0.172619). UCRP was cost-saving in 58.5% of simulations and QALY-gaining in 78.9%. At €30,000/QALY, UCRP was cost-effective in 78.3% of simulations versus 21.7% for SoC (probability of cost-effectiveness: 74.7% at €10,000/QALY, 77.8% at €20,000/QALY, 78.6% at €40,000/QALY, 78.7% at €50,000/QALY).

## Discussion

This early cost-effectiveness and headroom analysis of the UCRP in Italian ICUs estimated the conditions under which UCRP could provide favorable economic value compared with SoC over a 20-year time horizon. In the base-case analysis the model projected in UCRP to be dominant, driven by acute-phase ICU and hospital-ward savings. The detailed cost breakdown attributes €437.08 of the saving to ICU care and €58.97 to hospital ward care, against a small net increase in long-term Markov-phase costs (-€102.73) because more patients survive to accrue future healthcare costs. Results were driven primarily by ICU daily cost, AKI incidence, the modelled severity-shift effect, and UCRP diagnostic sensitivity, and changed materially under incidence, effect-size, and diagnostic-accuracy scenarios. These estimates are conditional on linked evidence and modelling assumptions and should not be interpreted as observed clinical or economic effects.

The sensitivity and threshold analyses identified AKI incidence, alongside ICU daily cost and the severity-shift effect, as the most influential drivers of the result. The model remains cost-saving down to an incidence of 4.84% and NMB-positive down to 1.05%, which provides some reassurance that the qualitative conclusion is not fragile to this assumption, but the magnitude of the projected benefit scales directly with incidence and should not be generalized beyond the deployment population it is intended to represent.

This analysis has several limitations that should be considered when interpreting the results. The UCRP intervention cost is included in every reported result, but its value is commercially confidential and is not disclosed; the separately reported headroom figure (€1,877.16 per patient) is a theoretical ceiling, not the manufacturer’s actual price. The diagnostic accuracy of UCRP (90% sensitivity, 70% specificity) is internal manufacturer data rather than a published estimate; the published Italian external-validation accuracy (82.5%/83.2%) is examined as a scenario and is somewhat less favorable to UCRP. Evidence for the clinical effect is limited to a single-centre, uncontrolled, postsurgical before-and-after implementation study (12/103 versus 9/99 moderate/severe AKI), which does not establish a causal effect and has a wide plausible range [18]. Long-term Markov transition probabilities were sourced from the UK NICE HTA [15], as Italian-specific long-term renal progression data of sufficient quality were not available. The model adopts a healthcare payer perspective and does not capture broader societal costs, including productivity losses or informal caregiver burden, meaning the true economic benefit of UCRP may be underestimated. The illustrative 15-bed allocation used for per-bed results is a linear cost-allocation convention, not a calibrated occupancy or capacity model, and per-bed figures should not be read as evidence of achievable throughput. Potential learning effects, changes in clinical practice following UCRP adoption, and capacity benefits arising from reduced ICU occupancy are not modelled. Renal transplantation was excluded because, under conditions of deceased-donor kidney scarcity in Italy, transplantation may largely redistribute waiting time and dialysis burden across patients; however, omitting recipient QALY gains, avoided dialysis costs, graft-related costs, and waiting-list displacement means that the net system-level effect cannot be assumed to be exactly neutral. Cost inputs were drawn from sources with price years ranging from 2016 to 2020 and were not adjusted to a common year, which may introduce modest uncertainty in the reported cost estimates.

Several areas warrant further investigation. Prospective clinical studies in Italian ICUs are needed to validate both the diagnostic performance of UCRP and the magnitude of AKI severity downstaging achievable with early KDIGO-guided intervention. Collection of Italy-specific long-term renal progression data would strengthen the generalizability of the economic analysis. Extending the model to a societal perspective and adapting the framework to other European healthcare systems would further test the robustness and transferability of these findings. Finally, real-world implementation studies examining the operational impact of UCRP on ICU workflow and clinician adherence to KDIGO bundles would provide evidence on benefits that the current model does not capture.

## Conclusions

In this early cost-effectiveness and headroom analysis, UCRP was projected to be dominant relative to SoC in Italian ICU settings under base-case assumptions, saving an estimated €363.42 per patient (€726,832 per 2,000-patient cohort) while generating an additional 0.049708 discounted QALYs per patient and 8.6 additional survivors per cohort annually, for a net monetary benefit of €1,854.66 per patient at €30,000/QALY. These results were driven predominantly by acute-phase ICU and hospital-ward savings rather than long-term CKD/ESRD prevention with savings ranging from €135.93 to €601.21 per patient. The headroom analysis for UCRP price estimated the maximum justifiable price at €1,877.16 per patient for UCRP.

These findings estimate the conditions under which UCRP could provide economic value; they do not demonstrate real-life clinical benefit, cost savings or operational capacity gains. The modelled clinical effect is based on a single, small, uncontrolled, non-causal implementation study, and diagnostic accuracy in the base case is internal manufacturer data rather than published external validation. Independent prospective multicenter validation of diagnostic performance, clinical effect, and implementation costs in Italian ICU populations is required before these results should inform adoption decisions.

**Table 1:** Discounted cost-effectiveness results for 20-years

Outcome	SoC	UCRP	Saving/gain in favor of UCRP (UCRP vs. SoC)	Per ICU bed (*n* = 15)
**Per-patient results**				
Total discounted cost (€)	38,059.95	37,696.53	363.42	-
Total discounted QALYs	9.890	9.940	0.049708	-
ICU cost (€)	7,078.60	6,641.52	437.08	-
Hospital ward cost (€)	7,562.34	7,503.37	58.97	-
Home cost (€)	1,708.74	1,716.14	-7.40	
Long-term cost (€)	21,710.27	21,813.00	-102.73	-
ICU days	4.22	3.95	0.27	-
Ward days	15.89	15.76	0.12	-
90-day mortality	13.72%	13.29%	0.43%	-
Net monetary benefit (€, at €30,000/QALY)	-	-	1,854.66	-
ICER (€/QALY)	-	-	Dominant	-
**Cohort results (per 2**,**000 patients)**				
Total discounted cost (€)	76,119,898	75,393,066	726,832	48,455.47
Total discounted QALYs	19,780.4	19,879.8	99.4	6.627758
ICU cost (€)	14,157,207	13,283,043	874,164	58,277.61
ICU + Hospital cost (€)	29,281,880	28,289,784	992,096	66,139.74
ICU days	8,435.2	7,897.6	537.6	35.84
90-day survivors	1,725.6	1,734.2	8.6	-

Positive values in the “Saving/gain in favor of UCRP” column indicate savings or QALY gains for UCRP; negative values indicate savings or QALY gains for SoC. Per-bed results use a linear allocation convention of 133.33 modelled patients per bed/year (see Methods) and are not a calibrated capacity or throughput estimate

## Supplementary Information

Below is the link to the electronic supplementary material.

Supplementary Material 1

## Data Availability

Data are available at the corresponding author upon reasonable request.

## Abbreviations

AKI: Acute kidney injury
CKD: Chronic kidney disease
ESRD: End-stage renal disease
FN: False negative
FP: False positive
ICU: Intensive care unit
SoC: Standard of care
TN: True negative
TP: True positive
UCRP: U-Care Renal Platform
