# Supplementary material for: Early cost-effectiveness analysis of the U-Care Renal Platform
Source: BMC Nephrol. 2026 Aug 14;27:666. doi: 10.1186/s12882-026-05289-y (PMC13753105; doi:10.1186/s12882-026-05289-y)
Supplement: Supplementary file 1 [file 12882_2026_5289_MOESM1_ESM.docx]

# Appendix

Incremental cost per patient (€)

| Incremental QALYs per patient | PSA iterations |
| --- | --- |
| 0.0728604 | -1559.95 |
| 0.0426162 | -590.802 |
| 0.0681909 | -455.472 |
| 0.0725821 | -846.865 |
| -0.0586273 | 158.999 |
| -0.0234139 | 354.579 |
| 0.16423 | -1044.1 |
| -0.0120162 | -14.5206 |
| 0.126641 | -3229.76 |
| 0.0784916 | -831.064 |
| 0.0864313 | -309.071 |
| 0.0637614 | -148.217 |
| 0.0231827 | 17.3582 |
| -0.0494691 | 197.635 |
| 0.0926416 | -459.798 |
| 0.00245282 | -0.237944 |
| 0.0708682 | -42.026 |
| 0.152984 | -1387.36 |
| -0.0172399 | 129.223 |
| 0.031266 | -152.914 |
| -0.272677 | 3375.17 |
| 0.116252 | -1523.43 |
| 0.0203771 | 164.413 |
| -0.0165994 | 85.6008 |
| 0.119336 | -259.394 |
| -0.024245 | 109.262 |
| 0.131259 | 1498.96 |
| 0.187204 | -687.515 |
| 0.0874381 | 233.835 |
| 0.0548576 | 134.671 |
| -0.0579927 | -193.527 |
| 0.0777804 | -246.428 |
| -0.0826946 | 349.563 |
| 0.0318279 | 1323.66 |
| 0.132643 | -161.574 |
| 0.0832752 | 821.813 |
| 0.0805899 | 137.827 |
| -0.0278009 | 879.08 |
| 0.0380503 | -158.335 |
| 0.0575807 | -487.346 |
| 0.0767728 | -893.098 |
| 0.0552286 | -31.9207 |
| -0.0749817 | 331.373 |
| 0.00533678 | 12.1235 |
| 0.0552909 | 273.594 |
| 0.0559494 | -194.364 |
| 0.0970234 | 437.243 |
| 0.0828479 | -230.342 |
| 0.0480297 | -297.859 |
| 0.046943 | -148.004 |
| 0.0338243 | 100.682 |
| -0.0654407 | -200.593 |
| -0.240988 | 804.639 |
| 0.0649864 | -457.714 |
| 0.0165547 | 98.0161 |
| 0.0765357 | -363.837 |
| 0.0614882 | -41.3317 |
| -0.00963896 | -71.916 |
| 0.17834 | -1564.37 |
| -0.12147 | 2673.69 |
| 0.146002 | -796.962 |
| 0.185916 | 383.148 |
| 0.0965879 | -2817.32 |
| 0.111952 | -288.14 |
| -0.0521079 | -638.108 |
| 0.0861333 | 1218.68 |
| 0.0479864 | 75.2955 |
| 0.0845229 | -488.442 |
| -0.0229666 | 267.866 |
| 0.0932917 | -103.488 |
| 0.0736678 | -412.982 |
| -0.0460608 | 275.726 |
| 0.0590131 | -53.7037 |
| 0.130607 | 102.237 |
| 0.0369476 | -94.3707 |
| 0.106481 | -415.596 |
| -0.188674 | -51.263 |
| 0.0804907 | -403.848 |
| -0.0209001 | 373.749 |
| 0.00977103 | -45.5417 |
| 0.076458 | -924.61 |
| 0.0927935 | -515.364 |
| 0.092201 | 46.3948 |
| 0.0524827 | -364.134 |
| 0.0961476 | -165.495 |
| 0.0752976 | -257.175 |
| 0.11557 | 1650.87 |
| 0.0233252 | -83.4458 |
| 0.0753641 | 431.12 |
| 0.119474 | -958.346 |
| 0.0803626 | -220.355 |
| 0.0179664 | 9.9651 |
| 0.0241844 | 41.435 |
| -0.0017893 | 68.4736 |
| -0.103533 | 463.161 |
| 0.084363 | -1193.37 |
| -0.0688324 | 105.536 |
| 0.0213493 | -869.948 |
| 0.0958449 | -1143.12 |
| 0.00558682 | -103.713 |
| -0.135037 | 1146.19 |
| 0.141888 | 174.677 |
| -0.105148 | 1191.31 |
| 0.069433 | -381.607 |
| 0.0737991 | -689.081 |
| -0.0333786 | -114.449 |
| 0.0667809 | -426.616 |
| 0.0372249 | -346.143 |
| 0.0111723 | 180.103 |
| -0.0278801 | 603.889 |
| 0.101051 | -2481.93 |
| -0.00250883 | 44.6477 |
| 0.069412 | -1678.37 |
| 0.0625429 | -331.714 |
| 0.0721289 | 82.7642 |
| 0.0951237 | -1896.86 |
| -0.0626733 | 509.46 |
| 0.0820499 | -47.065 |
| 0.0837212 | -169.871 |
| -0.0119424 | 60.6581 |
| 0.0597692 | -616.568 |
| 0.0938326 | 916.295 |
| 0.139391 | -313.557 |
| 0.0035623 | 24.2688 |
| 0.106529 | -608.503 |
| 0.171565 | 673.721 |
| 0.0310157 | 94.5251 |
| -0.0127825 | 224.19 |
| 0.115108 | -2435.14 |
| 0.0286002 | -146.777 |
| 0.145544 | 315.113 |
| -0.0513367 | 602.762 |
| -0.000654274 | 38.9188 |
| 0.0950261 | -1211.24 |
| 0.0601097 | 797.994 |
| -0.00668361 | -7.48579 |
| 0.159162 | -287.86 |
| -0.100537 | -2709.06 |
| 0.0628666 | 698.179 |
| 0.00359934 | 16.578 |
| -0.0393623 | 304.209 |
| 0.0148059 | 112.033 |
| 0.154301 | 1260.1 |
| 0.0915596 | -245.996 |
| 0.0721359 | -838.863 |
| -0.0483058 | 434.257 |
| 0.16476 | 82.2552 |
| 0.0665465 | -51.9069 |
| -0.18276 | 1699.6 |
| 0.00469495 | -63.6699 |
| 0.0707444 | -531.455 |
| 0.139451 | 619.949 |
| -0.0989006 | -271.139 |
| 0.067364 | -267.033 |
| 0.0692598 | -258.482 |
| 0.0807824 | -216.64 |
| 0.13986 | 1369.2 |
| 0.149913 | 776.873 |
| -0.0108889 | 39.5443 |
| 0.153833 | -307.404 |
| 0.0339492 | 128.112 |
| 0.0729642 | -789.767 |
| 0.058635 | -47.4044 |
| 0.0894909 | -1087.4 |
| -0.0323551 | -477.256 |
| 0.184288 | 542.833 |
| 0.00978717 | -7.2552 |
| 0.00658798 | -1.38439 |
| 0.0163575 | 34.7518 |
| 0.00983742 | -94.2483 |
| 0.0289105 | -148.999 |
| 0.105798 | -631.453 |
| 0.0781496 | -1008.36 |
| 0.081328 | -986.007 |
| 0.0314522 | 352.77 |
| 0.148407 | 1028.33 |
| 0.0647319 | -1551.27 |
| 0.0486279 | -304.806 |
| 0.0679902 | -511.543 |
| 0.0352653 | -76.8895 |
| 0.0615309 | 113.238 |
| 0.0797788 | -28.7952 |
| 0.192988 | 1911.59 |
| -0.000712515 | 52.0344 |
| 0.129139 | 134.777 |
| 0.068306 | -75.2441 |
| 0.0257237 | 5.83905 |
| 0.0977283 | 2286.82 |
| 0.123762 | -757.65 |
| 0.0505259 | 92.1591 |
| 0.147257 | 1103.87 |
| -0.206044 | 2302.24 |
| -0.0281386 | 232.393 |
| 0.165776 | -865.854 |
| 0.0368175 | 479.983 |
| -0.0272962 | -82.1191 |
| 0.0469484 | -875.917 |
| 0.0905623 | -2210.63 |
| 0.085827 | -452.187 |
| 0.0628152 | -354.756 |

| Incremental QALYs per patient | Mean: +0.049 QALYs, -€175 |
| --- | --- |
| 0.0488298 | -175.468 |

Supplementary Figure S1. Cost-effectiveness plane.

Abbreviations: QALY, quality-adjusted life year.

Probability cost-effective

| Willingness-to-pay threshold (€ per QALY) | UCRP (Treated) | Standard of Care |
| --- | --- | --- |
| 0K | 58.49% | 41.51% |
| 1K | 61.16% | 38.84% |
| 2K | 63.64% | 36.36% |
| 3K | 65.93% | 34.07% |
| 4K | 68% | 32% |
| 5K | 69.67% | 30.33% |
| 6K | 70.99% | 29.01% |
| 7K | 72.24% | 27.76% |
| 8K | 73.03% | 26.97% |
| 9K | 73.87% | 26.13% |
| 10K | 74.68% | 25.32% |
| 11K | 75.26% | 24.74% |
| 12K | 75.84% | 24.16% |
| 13K | 76.15% | 23.85% |
| 14K | 76.57% | 23.43% |
| 15K | 76.81% | 23.19% |
| 16K | 77.11% | 22.89% |
| 17K | 77.37% | 22.63% |
| 18K | 77.48% | 22.52% |
| 19K | 77.59% | 22.41% |
| 20K | 77.75% | 22.25% |
| 21K | 77.84% | 22.16% |
| 22K | 77.92% | 22.08% |
| 23K | 77.95% | 22.05% |
| 24K | 78.08% | 21.92% |
| 25K | 78.13% | 21.87% |
| 26K | 78.22% | 21.78% |
| 27K | 78.22% | 21.78% |
| 28K | 78.29% | 21.71% |
| 29K | 78.3% | 21.7% |
| 30K | 78.31% | 21.69% |
| 31K | 78.37% | 21.63% |
| 32K | 78.4% | 21.6% |
| 33K | 78.43% | 21.57% |
| 34K | 78.46% | 21.54% |
| 35K | 78.52% | 21.48% |
| 36K | 78.53% | 21.47% |
| 37K | 78.54% | 21.46% |
| 38K | 78.56% | 21.44% |
| 39K | 78.57% | 21.43% |
| 40K | 78.58% | 21.42% |
| 41K | 78.56% | 21.44% |
| 42K | 78.58% | 21.42% |
| 43K | 78.59% | 21.41% |
| 44K | 78.6% | 21.4% |
| 45K | 78.62% | 21.38% |
| 46K | 78.62% | 21.38% |
| 47K | 78.65% | 21.35% |
| 48K | 78.67% | 21.33% |
| 49K | 78.67% | 21.33% |
| 50K | 78.68% | 21.32% |

Supplementary Figure S2. Cost-effectiveness acceptability curve.

Abbreviations: QALY, quality-adjusted life year; SoC, standard of care; UCRP, U-Care Renal Platform; WTP, willingness to pay.

Supplementary Table S1. Model input parameters.

| **Category** | **Parameter** | **Base case** | **PSA distribution** | **Distribution parameters** | **OWSA range** | **Source** |
| --- | --- | --- | --- | --- | --- | --- |
| Epidemiology | AKI incidence | 40.00% | Beta | α=59.60; β=89.40 (SE 0.04) | 32.0-48.0% | Manufacturer data |
| Epidemiology | Background CKD prevalence at model entry | 11.05% | Beta | α=5,932.86; β=47,758.14 (N=53,691) | 8.84-13.26% | [1] |
| Screening | UCRP sensitivity | 90.00% | Beta | α=54.42; β=6.05 (SE 0.0383) | 72.0-100% | Manufacturer data |
| Screening | UCRP specificity | 70.00% | Beta | α=55.77; β=23.90 (SE 0.0510) | 56.0-84.0% | Manufacturer data |
| AKI severity (SoC) | Stage 1 | 68.70% | Dirichlet | α=2,963.72 | — | [1] |
| AKI severity (SoC) | Stage 2 | 19.40% | Dirichlet | α=836.92 | 15.52-23.28% | [1] |
| AKI severity (SoC) | Stage 3 | 5.95% | Dirichlet | α=256.68 | 4.76-7.14% | [1] |
| AKI severity (SoC) | Stage 3 + RRT | 5.95% | Dirichlet | α=256.68 | 4.76-7.14% | [1] |
| Treatment effect | Severity shift, Stages 2/3/3+RRT in true positives | 22.00% | Lognormal | RR=0.78; log-SE=0.4179 (12/103 vs 9/99) | 17.6-26.4% | [2] |
| Clinical | 90-day mortality, no AKI | 4.90% | Beta | α=47.91; β=929.85 (SE 0.0069) | 3.92-5.88% | [1] |
| Clinical | 90-day mortality, AKI Stage 1 | 21.50% | Beta | α=29.92; β=109.25 (SE 0.0347) | 17.20-25.80% | [1] |
| Clinical | Mortality multiplier, Stage 2 | 1.602 | Lognormal | μ=0.4692; σ=0.0640 | 1.282-1.922 | [1] |
| Clinical | Mortality multiplier, Stage 3 | 2.151 | Lognormal | μ=0.7640; σ=0.0624 | 1.721-2.581 | [1] |
| Length of stay - ICU | No AKI | 2.1 | Lognormal | μ=0.0488; σ=1.1774 | 1.68-2.52 | [1] |
| Length of stay - ICU | Stage 1 | 4 | Lognormal | μ=0.6931; σ=1.1774 | 3.2-4.8 | [1] |
| Length of stay - ICU | Stage 2 | 8 | Lognormal | μ=1.3863; σ=1.1774 | 6.4-9.6 | [1] |
| Length of stay - ICU | Stage 3 | 26 | Lognormal | μ=2.5649; σ=1.1774 | 20.8-31.2 | [1] |
| Length of stay - ICU | Stage 3 + RRT | 26 | Lognormal | μ=2.5649; σ=1.1774 | 20.8-31.2 | [1] |
| Length of stay - ward | No AKI | 8.1 | Lognormal | μ=1.0986; σ=1.4094 | 6.48-9.72 | [1] |
| Length of stay - ward | Stage 1 | 26.3 | Lognormal | μ=2.6391; σ=1.1230 | 21.04-31.56 | [1] |
| Length of stay - ward | Stage 2 | 32.4 | Lognormal | μ=2.8904; σ=1.0842 | 25.92-38.88 | [1] |
| Length of stay - ward | Stage 3 | 28.4 | Lognormal | μ=2.8332; σ=1.0131 | 22.72-34.08 | [1] |
| Length of stay - ward | Stage 3 + RRT | 45 | Lognormal | μ=3.2935; σ=1.0131 | 36-54 | [1] |
| Costs - acute (€) | ICU day | 1,660.00 | Gamma | shape=25.00; scale=66.40 | 1,328-1,992 | [1] |
| Costs - acute (€) | Hospital ward day | 475.86 | Gamma | shape=25.00; scale=19.03 | 380.69-571.03 | [3] |
| Costs - acute (€) | Single RRT session | 128.95 | Gamma | shape=25.00; scale=5.16 | 103.16-154.74 | [4] |
| Costs - acute (€) | KDIGO care bundle | 91.69 | Gamma | shape=100.00; scale=0.92 | 73.35-110.03 | [1] |
| Costs - long-term (€) | CKD, annual | 2,474.00 | Gamma | shape=25.00; scale=98.96 | 1,979.20-2,968.80 | [5] |
| Costs - long-term (€) | ESRD without dialysis, annual | 5,229.80 | Gamma | shape=1.58; scale=3,310.95 | 4,183.84-6,275.76 | [5] |
| Costs - long-term (€) | ESRD with dialysis, annual | 44,322.00 | Gamma | shape=25.00; scale=1,772.88 | 35,457.60-53,186.40 | [4] |
| AKI-to-CKD conversion | No AKI → CKD | 0.44% | Beta | α=214.16; β=48,458.62 (SE 0.0003) | 0.352-0.528% | [1] |
| AKI-to-CKD conversion | AKI Stage 1 → CKD | 0.88% | Beta | α=213.21; β=24,015.12 (SE 0.0006) | 0.704-1.056% | [1] |
| AKI-to-CKD conversion | AKI Stage 2 → CKD | 1.32% | Beta | α=212.26; β=15,867.93 (SE 0.0009) | 1.056-1.584% | [1] |
| AKI-to-CKD conversion | AKI Stage 3 and 3+RRT → CKD | 1.76% | Beta | α=211.31; β=11,794.80 (SE 0.0012) | 1.408-2.112% | [1] |
| Markov transitions | Outpatient → CKD | 0.44% | Beta | α=214.16; β=48,458.62 (SE 0.0003) | 0.352-0.528% | [1] |
| Markov transitions | CKD → remain / death / ESRD / ESRD+dialysis | 0.9200 / 0.0300 / 0.0100 / 0.0400 | Dirichlet | α=(8,280.00, 270.00, 90.00, 360.00) | ±20% per transition | [1] |
| Markov transitions | ESRD → remain / death / ESRD+dialysis | 0.7000 / 0.1200 / 0.1800 | Dirichlet | α=(3,500.00, 600.00, 900.00) | ±20% per transition | [1] |
| Markov transitions | ESRD + dialysis → death | 15.00% | Beta | α=764.85; β=4,334.15 (SE 0.005) | 12.00-18.00% | [1] |
| Utilities | ICU (acute) | 0.4052 | Beta | α=243.74; β=357.79 (SE 0.0200) | 0.3242-0.4862 | [1] |
| Utilities | Hospital ward (acute) | 0.44 | Beta | α=161.18; β=205.14 (SE 0.0259) | 0.3520-0.5280 | [1] |
| Utilities | Home (acute, post-discharge) | 0.62 | Beta | α=202.75; β=124.27 (SE 0.0268) | 0.4960-0.7440 | [1] |
| Utilities | Outpatient (no CKD, long-term) | 0.666 | Fixed | Age-adjusted background utility | - | [1] |
| Utilities | CKD disutility | −0.1100 | Beta | α=26.81; β=216.94 (SE 0.02) | −0.0880 to −0.1320 | [1] |
| Utilities | ESRD disutility | −0.3200 | Beta | α=43.20; β=91.80 (SE 0.04) | −0.2560 to −0.3840 | [1] |
| Utilities | ESRD + dialysis disutility | −0.4100 | Beta | α=39.26; β=56.50 (SE 0.05) | −0.3280 to −0.4920 | [1] |

# References

1. Brazzelli M, Aucott L, Aceves-Martins M, Robertson C, Jacobsen E, Imamura M, et al. Biomarkers for assessing acute kidney injury for people who are being considered for admission to critical care: a systematic review and cost-effectiveness analysis. Health Technol Assess. 2022;26:1–286. https://doi.org/10.3310/UGEZ4120.

2. Alfieri F, Ancona A, Bacci A, Zappalà S, Perez JM. Impact of an Artificial Intelligence–Powered Clinical Decision Support System for Acute Kidney Injury Prevention in the Intensive Care Unit: Single-Center Uncontrolled Before-and-After Implementation Study. JMIR Form Res. 2026;10:e87738–e87738. https://doi.org/10.2196/87738.

3. Foglia E, Ferrario L, Schettini F, Pagani MB, Dalla Bona M, Porazzi E. COVID-19 and hospital management costs: the Italian experience. BMC Health Serv Res. 2022;22:991. https://doi.org/10.1186/s12913-022-08365-9.

4. Bellasi A, Di Iorio B, Di Lullo L. Kidney Diseases: Challenges and Opportunities of the Third Millenium. How can digital health help the National Health System? AboutOpen. 2020;7:1–3. https://doi.org/10.33393/abtpn.2020.2029.

5. Turchetti G, Bellelli S, Amato M, Bianchi S, Conti P, Cupisti A, et al. The social cost of chronic kidney disease in Italy. Eur J Health Econ HEPAC Health Econ Prev Care. 2017;18:847–58. https://doi.org/10.1007/s10198-016-0830-1.
